# Anxiety and associated factors in Northwest Ethiopian pregnant women: a broad public health concern

**DOI:** 10.3389/fpubh.2023.1300229

**Published:** 2024-01-08

**Authors:** Tsion Tadesse Haile, Azmeraw Ambachew Kebede, Dereje Nibret Gessesse, Nuhamin Tesfa Tsega, Mastewal Belayneh Aklil, Wubedle Zelalem Temesgan, Tazeb Alemu Anteneh, Nebiyu Solomon Tibebu, Haymanot Nigatu Alemu, Asmra Tesfahun Seyoum, Agumas Eskezia Tiguh, Ayenew Engida Yismaw, Muhabaw Shumye Mihret, Goshu Nenko, Kindu Yinges Wondie, Birhan Tsegaw Taye, Marta Yimam Abegaz

**Affiliations:** ^1^Department of General Midwifery, School of Midwifery, College of Medicine and Health Sciences, University of Gondar, Gondar, Ethiopia; ^2^Department of Clinical Midwifery, School of Midwifery, College of Medicine and Health Sciences, University of Gondar, Gondar, Ethiopia; ^3^Department of Women’s and Family Health, School of Midwifery, College of Medicine and Health Sciences, University of Gondar, Gondar, Ethiopia; ^4^Department of Psychiatry, College of Medicine and Health Sciences, University of Gondar, Gondar, Ethiopia; ^5^School of Nursing and Midwifery, Asrat Woldeyes Health Science Campus, Debre Berhan University, Debre Berhan, Ethiopia

**Keywords:** anxiety, pregnancy, women, factors, Gondar city

## Abstract

**Introduction:**

Pregnancy-related anxiety is a prevalent mental health issue that mostly affects women in low-income countries such as Ethiopia. It has been linked to unfavorable pregnancy outcomes, such as miscarriage, prematurity, and low birth weight. However, it has often received less attention, and community-based evidence lacks its prevalence and associated factors. Thus, the purpose of this study was to assess the prevalence and associated factors of anxiety in Northwest Ethiopian pregnant women in Gondar city.

**Methods:**

A community-based cross-sectional study was conducted from 1 July to 30 August 2021 in Gondar city. A cluster sampling technique was used to select a sample of 872 pregnant women, and in-person interviews were conducted to gather data. Descriptive and analytical statistical procedures were carried out.

**Results:**

Of the participants, pregnancy-related anxiety was reported in 29.4% (95% CI: 26.3, 32.4) of women. The likelihood of having anxiety was higher among women who had known medical illness (AOR = 3.16; 95% CI: 1.8, 5.35), loneliness (AOR = 2.52; 95% CI: 1.34, 4.73), depression (AOR = 2.38; 95% CI: 1.48, 3.85), poor social support (AOR = 1.93; 95% CI: 1.21, 3.07), and intimate partner violence (AOR = 2.87; 95% CI: 2.04, 4.04).

**Conclusion:**

In this study, three out of ten women have suffered from anxiety. It is strongly advised to identify and treat known medical illnesses early in pregnancy, enhance social support, diagnose and treat depression, and limit intimate partner violence through multimodal and integrative activities with concerned bodies.

## Introduction

For the majority of women and their families, pregnancy is a time of immense joy, happiness, contentment, and self-fulfillment. Nonetheless, a sizeable portion of women may experience unfavorable circumstances related to their physical and mental health during pregnancy (1). This negative emotional state associated with concern about one’s own health and wellbeing, the baby’s health, and the coming childbirth process is called pregnancy-related anxiety (PRA) (2, 3). According to studies, anxiety is a significant complication that has been reported to impact 20–40% of women during pregnancy (4, 5).

Mental health problems, particularly PRA, are a neglected global public health problem, affecting the health of pregnant women and unborn babies (6). Approximately 10% of pregnant women worldwide have experienced mental health conditions, with anxiety being the most prevalent. It was one of the most common severe mental disturbances, impacting millions of individuals (7). Approximately 10 and 25% of pregnant women have experienced PRA in developed and developing countries, respectively (8, 9).

According to empirical evidence, PRA has been linked to a higher probability of adverse birth outcomes, particularly in developing countries (10), because women in resource-limited countries have limited access to prenatal and mental health services. Negative pregnancy outcomes associated with PRA include spontaneous abortion, preeclampsia, prematurity, low birth weight, fetal growth restriction, prolonged labor, and a higher incidence of cesarean section (11–13).

The existing literature has revealed that the prevalence of PRA is inconsistent across countries. Accordingly, studies have shown that the prevalence of PRA was 55.7% in southern India (2), 26.8% in Brazil (14), 70% in Pakistan (6), and 26.5% in Qatar (15). In addition, a study conducted in Nepal found that 40.9% of women had minimal anxiety, 42.1% had mild-to-moderate levels of anxiety, and 16.9% had severe anxiety (16). According to a different trimester-specific study conducted in Spain, the prevalence of anxiety was found to be 19.5% in the first trimester, 16.8% in the second trimester, and 17.2% in the third trimester (17). Antenatal anxiety varies from 7.7% in Poland to 36.5% in Italy, according to a recently added comprehensive study of European women (18).

Evidence demonstrated that PRA was higher among women with psychiatric comorbidity, stressful events (19), social disadvantage, history of adverse pregnancy outcomes (20), previous history of mental illness, or history of psychiatric treatment during a previous pregnancy (21). Furthermore, a number of common contributing factors to PRA have been discovered, including younger maternal age, nulliparous status, not having a live birth, women’s role in household decision-making, domestic violence, lower socioeconomic level, limited social support, and depression (2, 3, 6, 15).

Mental health issues of pregnant women receive little attention, especially in Ethiopia, despite the terrible effects of PRA on the health of both the mother and child. In addition, there was not much evidence from the community on PRA. In this context, determining the factors that contribute to PRA and setting corrective actions will have great input to be in line with the global target of reducing the maternal mortality ratio to as low as 70 per 100,000 live births by 2030 (22). Therefore, this study aimed to assess the prevalence and associated factors of anxiety in Northwest Ethiopian pregnant women in Gondar city.

## Materials and methods

### Study area, design, and period

In Gondar city, a community-based cross-sectional study was carried out between 1 July and 30 August 2021. Gondar city is located in Central Gondar Zone, Amhara national regional state, and it is 750 km away from Addis Ababa, the capital city of Ethiopia. There are one governmental comprehensive specialized hospital, eight governmental health centers, one private primary hospital, one general hospital, and 22 health posts serving the population in and outside the city. Of the projected 432,191 people living in the city, 224,508 were women. Approximately 133,477 (30.88%) of them belonged to the reproductive age group (unpublished data).

### Study participants and eligibility criteria

All pregnant women who resided for at least 6 months in Gondar city were the source population, and of these pregnant women, those who resided in the selected clusters of Gondar city and were available during the data collection time were the study population. Those who were seriously ill, unable to provide a response, and resided in the area for less than 6 months before the data collection period were excluded from the study.

### Sample size determination and sampling procedure

A single population proportion formula was used to determine the sample size. Since there was no published data in Ethiopia on PRA, 50% was taken as the proportion (*p*) of PRA; by considering a 95% level of confidence and 5% margin of error, the sample size (*n*) was calculated as *n* $=\frac{{\left(Z\alpha /2\right)}^{2}p\left(1-p\right)}{d^{2}}$ $\frac{={\left(1.96\right)}^{2}0.5\left(1-0.5\right)}{\left(0.05\right)2}$ = 384

Then, a sample size of 845 has been estimated after taking into account a 10% non-response rate and a design effect of 2 (because of cluster sampling). From 22 kebeles in Gondar city, 7 kebeles (30% of the total kebeles) were selected randomly using a lottery method. A house-to-house visit was carried out in the selected clusters (kebeles) to find eligible women for the study. There were 872 eligible women in the chosen clusters.

### Operational definitions and measurements of variables

#### Pregnancy-related anxiety

Pregnant women who scored ≥13 on the Pregnancy-Related Anxiety Questionnaire-Revised (PRAQR) were classified as having pregnancy-related anxiety, positive, or anxious, with 10 questions to be scored out of 30 points (23).

#### Household decision-making power

The ability of women to act self-sufficiently on the household activities including their health, their children’s health, freedom of movement, and control over finance without needing permission from another person (24). A total of 8 questions were used and scored out of 27 points. The women’s responses were coded as 2 (if women decided independently), 1 (if women decided with their husbands), and 0 (if the decision was made by their husband or somebody else), and then, women who answered above the mean value were considered to have higher decision-making power (24, 25).

#### Social support

It was measured using the “Oslo-3 Social Support Scale (OSSS-3).” The three components on the scale are related to the number of close intimates the woman has, the degree of concern she feels from others, and how easy she believes it is to seek help from her neighbors. The degree of social support received scores ranging from 3 to 8 for “poor,” 9 to 11 for “moderate,” and 12 to 14 for “strong” (26).

#### Depression

Nine questions were intended to measure depression. Women were classified as depressive if they obtained a score of ≥10 on the “patient health questionnaire-9 (PHQ-9)” (27).

#### Intimate partner violence

Regardless of the legal status of the relationship with the current or past intimate partner, women were considered to have suffered intimate partner violence if they answered “yes” to any one of the ranges of sexual, psychological, or physical acts or any combination of the three (28).

### Data collection tools, quality control, and data collectors

The tool was adapted and designed by reviewing different types of relevant literature (2, 3, 14). Face-to-face interviews with a structured questionnaire were used to gather the data. The questionnaire addresses sociodemographic characteristics, husband engagement, social support, decision-making autonomy, obstetric and maternal health service-related features, and questions measuring anxiety connected to pregnancy. The tool was initially created in English, translated into Amharic, the local language, for the purpose of gathering data, and then again translated into English for analysis, uniformity, and general understanding. A pretest was conducted on 5% of the total sample size in a location other than the study sites prior to the actual data collection period to assess suitability, response, and linguistic clarity of the questionnaire. Fourteen skilled BSc midwives working under the guidance of four MSc midwives gathered the data.

### Statistical analysis

After the data were checked, coded, and added to Epidata version 4.6, it was exported to SPSS version 25 for additional cleaning and examination. To show the characteristics of the individuals, descriptive statistics were produced, such as mean, proportion, and frequency. Variables with a *p-*value of less than 0.25 were included in the multivariable logistic regression analysis to help adjust for potential confounders. Binary logistic regression was used to identify independent predictors. Variables in the multivariable logistic regression analysis were deemed statistically significant if their *p-*value for the odds ratio was less than 0.05 at a 95% confidence interval. To evaluate the fitness of the model, the Hosmer–Lemeshow test was used.

### Ethical consideration

The Institutional Review Board (IRB) of the University of Gondar granted the ethical permission for the study with a reference number of V/P/RCS/05/2710/2021. The study was carried out in compliance with the institutional guidelines and local laws. Every research participant provided informed consent.

## Results

### Sociodemographic characteristics of the respondents

Of 872 women who made up the sample, 858 were included in the analysis, yielding a 98.4% response rate. The median age of the study participants was 29 years old (IQR: 33, 26), with roughly 40.8% of them falling within the 26–30 year age range. Of the women, over two-fifths (43.3%) had completed a diploma or higher education. Of the survey participants, the majority (95.7%) of them were married, and 44.4% worked as housewives (Table 1).

**Table 1:** Sociodemographic characteristics of pregnant women in Gondar city, Northwest Ethiopia, 2021 (*n* = 858).

Variables	Frequency	Percentage (%)
**Age of women in years**		
≤20	23	2.7
21–25	160	18.6
26–30	350	40.8
>30	325	37.9
**Religion**		
Orthodox Christian	706	82.3
Muslim	107	12.5
Others (Protestant and Adventist)	45	5.2
**Current marital status**		
Married	821	95.7
Single	37	4.3
**Educational status of the women**		
No formal education	105	12.2
Primary education	140	16.3
Secondary education	242	28.2
Diploma and above	371	43.3
**Occupation of the women**		
Housewife	381	44.4
Merchant	105	12.2
Self-employed	99	11.5
Daily laborer	34	4.0
Government employee	239	27.9
**Husband’s educational status (*n* = 779)**		
No formal education	59	7.6
Primary	55	7.1
Secondary	156	20
Diploma and above	509	65.4
**Husband occupation (*n* = 779)**		
Government employee	353	45.3
Merchant	177	22.7
Self-employed	164	21.1
Daily labor	56	7.2
Student	29	3.7
**Average monthly income of the family**		
<2,500 ETB	116	13.5
≥2,500 ETB	742	86.5
**Family size**		
<4	243	28.3
≥4	615	71.7

### Reproductive and medical-related characteristics of the respondents

Over half (53.9%) of the women in this study had a parity status of 2 to 4. In their most recent pregnancy, the majority (97.3%) of research participants had at least one ANC visit. Approximately 2.2% of women had known psychiatric problems, and nearly half (48.6%) of them had experienced intimate partner violence in the most recent pregnancy (Table 2).

**Table 2:** Reproductive and maternity health service characteristics of pregnant women in Gondar city, Northwest Ethiopia, 2021 (*n* = 858).

Variables	Frequency	Percentage
**Parity**		
1	364	42.4
2–4	462	53.9
>4	32	3.7
**Had ANC follow-up**		
Yes	835	97.3
No	23	2.7
**Number of ANC follow-up (*n* = 835)**		
<4	320	38.3
≥4	515	61.7
**Was the most recent pregnancy planned?**		
Yes	745	86.8
No	113	13.2
**Was the most recent pregnancy supported by the husband and/or partner?**		
Yes	794	92.5
No	64	7.5
**Social support**		
Poor support	247	28.8
Moderate support	392	45.7
Strong support	219	25.5
**Women’s decision-making power**		
Higher	515	60.0
Lower	343	40.0
**Intimate partner violence**		
Yes	417	48.6
No	441	51.4
**Depression**		
Yes	96	11.2
No	762	88.8
**Loneliness**		
Yes	760	88.6
No	98	11.4
**Known medical disorder**		
Yes	86	10
No	772	90
**Known psychiatric problem**		
Yes	19	2.2
No	839	97.8
**Family with a psychiatric problem**		
Yes	79	9.2
No	779	90.8
**Adverse life events**		
Yes	208	24.2
No	650	75.8

### Prevalence and factors associated with pregnancy-related anxiety

It was reported that 29.4% (95% CI: 26.3, 32.4) of the study participants had pregnancy-related anxiety (PRA). To find the factors connected to PRA, bivariable and multivariable logistic regressions were used. The results of the multivariable analysis showed that PRA was significantly associated with known medical illness (AOR = 3.16; 95% CI: 1.87, 5.35), loneliness (AOR = 2.52; 95% CI: 1.34, 4.73), depression (AOR = 2.38; 1.48, 3.85), poor social support (AOR = 1.93; 1.21, 3.07), and intimate partner violence (AOR = 2.87; 2.04, 4.04) (Table 3).

**Table 3:** Bivariable and multivariable analyses of factors associated with pregnancy-related anxiety in Gondar city, Northwest Ethiopia, 2021.

Variables	Pregnancy-related anxiety		COR (95% CI)	AOR (95% CI)
	Yes	No		
**Age of women**				
≤20	8	15	1	1
21–25	59	101	1.09 (0.44, 2.74)	1.02 (0.38, 2.74)
26–30	102	248	0.77 (0.32, 1.88)	0.58 (0.22, 1.53)
>30	83	242	0.64 (0.26, 1.57)	0.40 (0.15, 1.10)
**Parity**				
1	90	274	0.42 (0.20, 0.88)	0.45 (0.18, 1.11)
2–4	148	314	0.60 (0.29, 1.29)	0.75 (0.33, 1.72)
>4	14	18	1	1
**Pregnancy planned**				
Yes	203	542	1	1
No	49	64	2.04 (1.36, 3.06)	1.19 (0.69, 2.09)
**Pregnancy Supported**				
Yes	226	568	1	1
No	26	38	1.72(1.02, 2.89)	0.80 (0.39, 1.63)
**Known medical illness**				
Yes	44	42	2.84 (1.80, 4.46)	3.16 (1.87, 5.35)**
No	208	564	1	1
**Decision-making power**				
Low	115	228	1.39 (1.03, 1.87)	1.06 (0.76, 1.49)
High	137	378	1	1
**Loneliness**				
Yes	237	523	2.50 (1.42, 4.44)	2.52 (1.34, 4.73)*
No	15	83	1	1
**Depression**				
Yes	56	40	4.04 (2.61, 6.26)	2.38 (1.48, 3.85)**
No	196	566	1	1
**Social support**				
Poor	96	151	2.21 (1.47, 3.32)	1.93 (1.21, 3.07)*
Moderate	107	285	1.30 (0.88, 1.92)	1.39 (0.91, 2.14)
High	49	170	1	1
**Intimate partner violence**				
Yes	177	240	3.59 (2.63, 4.93)	2.87 (2.04, 4.04)**
No	75	366	1	1

Bold: significantly associated, AOR: adjusted odds ratio, COR: crude odds ratio, CI: confidence interval, 1: reference category, and *p < 0.05;**p < 0.001.

## Discussion

Our study was aimed at assessing pregnancy-related anxiety (PRA) and associated factors. Thus, the prevalence of PRA was found to be 29.4%. The finding is consistent with research from Tanzania, 25% (23), and Brazil, 26.6% (14).

However, the finding of our study is lower than those of studies conducted in Benin (44.91%) (29), Nepal (46.4%) (30), India (55.7%) (2), and China (59.07%) (31). A plausible explanation for the disparity between research conducted in India and Nepal could be the variations in the sociodemographic attributes of study participants, such as age. Just 2.7% of study participants in this study were older than 20 years compared with almost 30 and 18.3% of study participants in studies conducted in India and Nepal, respectively. Evidence has demonstrated that younger maternal age is a significant contributing factor for PRA (3). The discrepancy with the study conducted in Benin might be the difference in the characteristics of study participants, such as the experience of adverse life events. Approximately 24.2% of the study participants in this study had unfavorable life events compared with 61.76% of the study participants in Benin. According to available evidence, women who have had unfavorable life events are more likely to develop anxiety (32, 33). Moreover, as gestational diabetes mellitus (GDM) affects both the physical and mental health of the mother and the unborn child, it may increase the prevalence of PRA. This is because the study conducted in China included all pregnant women with GDM (34).

Compared to other studies, the study’s result is higher than those of studies conducted in South Africa, 15.2% (35), West Africa, 11.4% (36), Saudi Arabia, 23.6% (37), Changchun China, 20.6% (38), and Southwestern China, 15.04% (39). The possible justification for this difference might be the discrepancy in the study population. This study was a community-based study that focused on both women who had ANC follow-up and had no ANC follow-up, while the studies conducted in South Africa, West Africa, Saudi Arabia, and Changchun China were hospital-based studies that focused only on women who had ANC follow-up. Because health promotion and disease prevention are one of the goals of ANC visit, women who had ANC follow-up might have adequate information related to the problem and may have good health-seeking practices that might decrease the prevalence of PRA; while some of the study participants in this study did not have ANC follow-up, there might be increased PRA (40). The discrepancy with the study conducted in southwestern China might be due to the difference in psychosocial characteristics of the study participants, such as depression and perceived low social support. Both factors are significantly associated risk factors of PRA, but 5.16 and 21.39% of the study participants in the study conducted in southwestern China had depression and perceived low social support, respectively, while it was slightly higher in this study, which was 11.2 and 28.8%. Since the study participants in this study were riskier, the prevalence of PRA might be higher (2).

This study found that the odds of having PRA among women who had known medical illnesses were three times higher than their counterparts (19). This might be due to the fact that PRA is a concern about the health of the mother, the fetus, and the pregnancy. Thus, medical illness might lead to mental health deterioration because of the fear of its complications on the fetus, the pregnancy, and the maternal condition herself. In addition to this, medical illness might decrease the social interaction of women and increase perceived isolation.

This study also found that women who had experienced loneliness were 2.5 times more likely to have PRA compared with those who had not experienced loneliness. This can be explained by the reason that loneliness is the sense of unwanted, isolation, and stigmatization that can bring the feeling of being unwell, something left, and separated; hence, there might be a fear about the pregnancy, one’s own health, and the fetus, which is PRA (41).

In this study, depression is also a significantly associated factor with PRA. Women who have experienced depression were 2.38 times more likely to have had PRA than women who were not depressed. This finding is supported by the studies conducted in India (2) and southwestern China (39). This might be due to the reason that increased depressive symptoms are associated with decreased engagement in favorable health practices during pregnancy, and it hampers ANC service utilization, which, in turn, results in low health information attainment and might increase the prevalence of PRA. The co-morbid existence of depression and anxiety was also observed in a study conducted in southern India (2).

Similarly, the odds of having PRA among women who have had perceived poor social support were 1.93 times higher than those who have had high social support. This finding is supported by studies conducted in India (2), China (39), and Pakistan (20). This might be due to the fact that social support is an informational tool and emotional support from family, friends, neighbors, and colleagues. As a result, lack of support or care from the abovementioned individuals can lead to a sense of remoteness, which, in turn, could rise to PRA (42).

This study also found that the odds of having PRA among women who have had intimate partner violence during their most recent pregnancy were 2.87 times higher than their counterparts. This finding is supported by a study conducted in Nigeria (43). The possible explanation might be that intimate partner violence is a harsh external factor that disturbs the physical, social, and emotional wellbeing of women. Hence, a sense of being offended and insignificant may bring a poor mental health state (44).

## Limitations

Since the study design is cross-sectional, it is possible that the true cause-and-effect relationship between the independent and dependent variables will be impacted. Furthermore, because of the nature of the cluster sampling technique, the calculated sample size did not match the actual sample size used for data collection and analysis.

## Conclusion

The prevalence of pregnancy-related anxiety was fairly high in the study area. It was significantly associated with known medical illnesses, loneliness, depression, poor social support, and intimate partner violence. There should be integrated and coordinated action by a multidisciplinary and interdisciplinary team to attain favorable physical and mental wellbeing of pregnant women by identifying and treating known medical illnesses early in pregnancy, thus improving social support, identifying and treating depression, and minimizing intimate partner violence.

Future researchers are recommended to carry out longitudinal studies and look at how other socioeconomic and political issues affect the mental health of pregnant women.

## Data availability statement

The raw data supporting the conclusions of this article will be made available by the authors, without undue reservation.

## Ethics statement

The study involving humans was approved by University of Gondar Institutional Review Board (IRB) (Reference number: V/P/RCS/05/2710/2021). The study was conducted in accordance with the local legislation and institutional requirements. The participants provided informed consent to participate in this study.

## Author contributions

TTH: Methodology, Writing – original draft, Writing – review & editing. AAK: Conceptualization, Investigation, Methodology, Writing – review & editing. DNG: Supervision, Writing – review & editing. NTT: Software, Writing – review & editing. MBA: Investigation, Writing – review & editing. WZT: Investigation, Writing – review & editing. TAA: Methodology, Writing – review & editing. NST: Investigation, Supervision, Writing – review & editing. HNA: Supervision, Writing – review & editing. ATS: Data curation, Supervision, Writing – review & editing. AET: Investigation, Writing – review & editing. AEY: Validation, Writing – review & editing. MSM: Conceptualization, Methodology, Validation, Writing – review & editing. GN: Data curation, Supervision, Writing – review & editing. KYW: Investigation, Writing – review & editing. BTT: Data curation, Writing – review & editing. MYA: Data curation, Formal analysis, Methodology, Writing – review & editing.
